# Vertebrate seed dispersers maintain the composition of tropical forest seedbanks

**DOI:** 10.1093/aobpla/plv130

**Published:** 2015-11-17

**Authors:** E. M. Wandrag, A. E. Dunham, R. H. Miller, H. S. Rogers

**Affiliations:** 1 Biosciences at Rice, Rice University, Houston, TX 77005, USA; 2 Institute for Applied Ecology, University of Canberra, Bruce, ACT 2617, Australia; 3 College of Natural and Applied Sciences, University of Guam, Mangilao, GU 96923, USA; 4 Department of Ecology, Evolution, and Organismal Biology, Iowa State University, Ames, IA 50011 USA

**Keywords:** Bird loss, community ecology, island ecology, mutualisms, plant recruitment, tropical forest ecology

## Abstract

The accumulation of seeds in the soil (the seedbank) can set the template for the early regeneration of habitats following disturbance. Seed dispersal is an important factor determining the pattern of seed rain, which affects the interactions those seeds experience. For this reason, seed dispersal should play an important role in structuring forest seedbanks, yet we know little about how that happens. Using the functional extirpation of frugivorous vertebrates from the island of Guam, together with two nearby islands (Saipan and Rota) that each support relatively intact disperser assemblages, we aimed to identify the role of vertebrate dispersers in structuring forest seedbanks. We sampled the seedbank on Guam where dispersers are absent, and compared this with the seedbank on Saipan and Rota where they are present. Almost twice as many species found in the seedbank on Guam, when compared with Saipan and Rota, had a conspecific adult within 2 m. This indicates a strong role of vertebrate dispersal in determining the identity of seeds in the seedbank. In addition, on Guam, a greater proportion of samples contained no seeds and overall species richness was lower than on Saipan. Differences in seed abundance and richness between Guam and Rota were less clear, as seedbanks on Rota also contained fewer species than Saipan, possibly due to increased post-dispersal seed predation. Our findings suggest that vertebrate seed dispersers can have a strong influence on the species composition of seedbanks. Regardless of post-dispersal processes, without dispersal, seedbanks no longer serve to increase the species pool of recruits during regeneration.

## Abstract (precis)

Seed dispersal is considered a key mechanism through which the structure and function of forests is maintained. Testing this can be difficult because the large scale over which dispersal operates makes it difficult to examine in a meaningful way. Using the near complete loss of native vertebrate seed dispersers from the island of Guam we examine the importance of seed dispersal for maintaining forest seedbanks. We find that seed dispersers have a strong influence on the species composition of seedbanks. Without seed dispersers seedbanks no longer serve to increase the species pool for tree regeneration following disturbance.

## Introduction

Seeds present in or on the soil (the soil seedbank) provide the template for plant recruitment and can be important for the regeneration of habitats immediately following disturbance (Chandrashekara and Ramakrishnan 1993; Grombone-Guaratini and Rodrigues 2002). Because the spatial pattern of seed deposition can influence the interactions that seeds are involved in, processes that affect those patterns could have important consequences for seed fate (Bigwood and Inouye 1988), with implications for plant population and community dynamics (Beckman and Rogers 2013). Frugivorous vertebrates are a dominant mechanism of seed dispersal in many ecosystems, especially tropical forests where between 70 and 94 % of tree species are estimated to rely on vertebrates for the dispersal of their seeds (Jordano 2000). However, while we increasingly understand how seed dispersers might shape patterns of seed distribution at the plant species level (e.g. Caughlin *et al*. 2015), we know little about how they may alter the distribution and local community structure of seeds in the seedbank.

For species that rely on vertebrate dispersers for the dispersal of their seeds, there are three key ways in which those dispersers might influence the structure and composition of the seedbank. First, dispersal moves seeds away from parent plants and should decrease the probability that seeds in the seedbank are in close proximity to a conspecific adult. This is important because natural enemies such as fungal pathogens and seed predators often concentrate close to parent plants (Wright 2002; Comita *et al*. 2014) and dispersal can thus reduce distance-dependent seed mortality associated with proximity to conspecifics (Janzen 1970; Connell 1971). Second, by redistributing seeds within the landscape, seed dispersal may alter the spatial aggregation of seeds, leaving fewer ‘seed gaps’ on the forest floor (e.g. seedless areas under non-fruiting trees) and reducing areas of high local seed density (i.e. under fruiting trees). A disadvantage of seedless patches within the seedbank would be a reduction in the availability of seeds for seedling regeneration following disturbance, and an increase in density-dependent mortality associated with clustered seed deposition patterns (Russo 2005). Third, by increasing the movement of seeds within the forest, vertebrate seed dispersers expand the available species pool for any given area and should thereby increase the local species richness of seeds present in the soil seedbank.

Understanding the role of vertebrate-mediated seed dispersal in structuring forest communities is increasingly important because vertebrate populations continue to decline from forests around the world (e.g. Savidge 1987; Terborgh *et al*. 2008). If the loss of vertebrate dispersers has ramifications for forest seedbanks, then this could have implications for forest regeneration and persistence. Nevertheless, few studies have attempted to identify the role of vertebrate dispersers in structuring forest seedbanks. One reason for this is that it is difficult to manipulate entire vertebrate assemblages at scales large enough to meaningfully identify their role at the community level. Loss of vertebrate species from forests by hunting or other anthropogenic pressures may provide one way in which to examine the role of vertebrate seed dispersal in structuring seedling communities (Terborgh *et al*. 2008; Effiom *et al*. 2013; Harrison *et al*. 2013). However, such forests are rarely completely free of vertebrate dispersers, and none of these studies have examined the seedbank.

We take advantage of an ‘accidental experiment’ (HilleRisLambers *et al*. 2013) provided by the functional extirpation of frugivorous vertebrates from the island of Guam to test predictions about the role of native vertebrate dispersers in determining both the distribution and local species composition of seeds of tree species in tropical forest seedbanks. The introduction of the brown tree snake, *Boiga irregularis* (Colubridae), to Guam in the 1940s resulted in the extinction of native bird species, including four of the five frugivorous birds that were previously present (Savidge 1987) **[see** **Supporting Information****—Table S1]**. The fifth frugivorous bird experienced extreme range contractions and is now functionally extinct from Guam's forests. In contrast, the nearby islands of Saipan (Camp *et al*. 2009) and Rota (Camp *et al*. 2014), which both support similar limestone forest to Guam, have a more intact disperser assemblage, though some populations in Rota are declining (Camp *et al*. 2014). In addition to frugivorous birds, there is one species of frugivorous bat native to the Mariana Islands (*Pteropus mariannus* Desmarest). However, this bat species is also now functionally extinct from forests on both Guam and Saipan, and present only on Rota in reduced abundance. These native dispersers have not been replaced by non-native species, with the potential exception of feral pigs (*Sus scrofa*, present on Guam and Rota but excluded from plots used in this study). Since ∼85 % of the tree species present in the Marianas have seeds adapted for dispersal by vertebrate frugivores (H. S. Rogers, unpubl. data), this system provides a rare opportunity to examine the consequences of the functional extirpation of native vertebrate dispersers for the seedbank.

We examined the distribution and composition of seeds in the seedbanks of forests with native frugivorous vertebrates (Saipan and Rota) when compared with those without (Guam). We predicted that if vertebrate seed dispersers are important for maintaining forest seedbanks, then we would see differences in the distribution and composition of the seedbank on Guam relative to Saipan and Rota. We hypothesized that the presence of frugivorous vertebrates on Saipan and Rota would be associated with greater seed movement that would result in (i) a greater proportion of species in the seedbank that lack nearby conspecific adults, (ii) a more regular distribution of seeds (i.e. fewer sites that are either devoid of seeds or have high seed densities) and (iii) higher species richness per seedbank sample.

## Methods

### Study area

**Figure 1.**
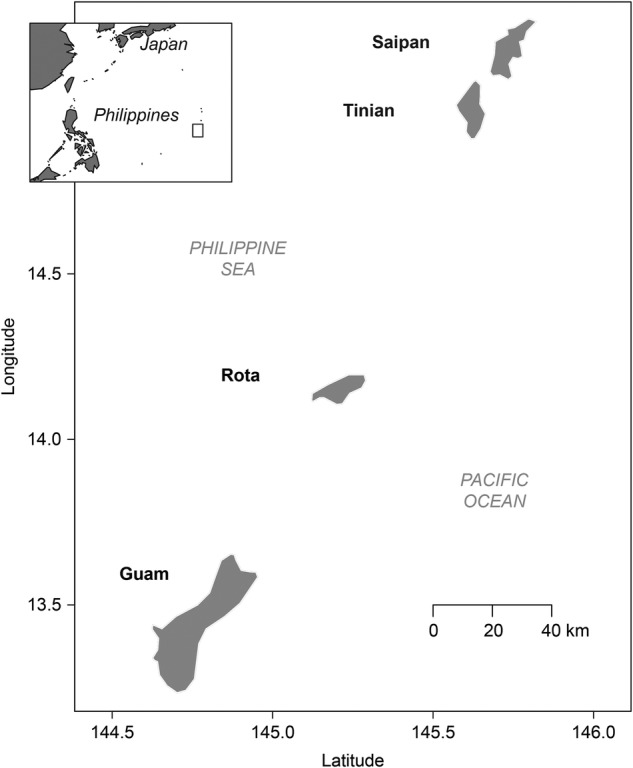
Map showing the location of Guam, Rota and Saipan.

The islands of Guam, Saipan and Rota are located within the Mariana Island chain, in the Western Pacific (Fig. 1). They have a mean annual temperature of ∼27 °C, with little seasonal variation. All islands experience frequent typhoons, which can cause considerable damage to vegetation (Kerr 2000), and pronounced wet and dry seasons.

We conducted our study within limestone forest, which overlies karst formed by uplifted coral plateaus. The forest is moist, broadleaved and evergreen (Mueller-Dombois and Fosberg 1998) and characterized by species such as *Aglaia mariannensis* (Meliaceae ); *Artocarpus mariannensis* (Moraceae); *Cynometra ramiflora* (Leguminosae); *Elaeocarpus joga* (Elaeocarpaceae); *Ficus prolixa* (Moraceae); *Meiogyne cylindrocarpa* (Annonaceae, previously known as *Guamia mariannae*); *Ochrosia mariannensis* and *O. oppositifolia* (Apocynaceae, previously known as *Neisosperma oppositifolia*); *Pandanus dubius* and *P. tectorius* (Pandanaceae); *Pisonia grandis* (Nyctaginaceae) and *Premna serratifolia* (Lamiaceae, previously known as *Premna obtusifolia*). A survey of this forest type in Saipan in 1992 recorded 27 tree species occupying the canopy, with a further 22 in the understorey (Craig 1992). The forest has a particularly short canopy with most trees <11 m tall (Donnegan *et al*. 2011), likely as a result of the frequent typhoons.

### Soil sampling

We collected soil samples between December 2013 and January 2014 to identify relative differences in the distribution and composition of the seedbank between islands. Although seedbank composition may vary seasonally (Dalling *et al*. 1997), we were primarily interested in identifying relative differences between islands at a single time point. Peak fruiting occurs between May and August in these islands, so we expect samples from December and January to be dominated by seeds waiting for an opportunity to germinate from the seedbank rather than recently fallen seeds.

We sampled within 44 plots spread across 11 forest sites on 3 islands. These sites were established between 2008 and 2009 as part of a long-term forest research project. There are five sites on Guam and three on each of Saipan and Rota. Within each of these sites, four plots ranging in size between 8 and 12 m^2^ were demarcated for a separate experiment. This gave a total of 20 plots sampled on Guam and 12 each on Saipan and Rota. Plots were at least 20 m apart and centred on at least one of three common forest species (*A. mariannensis*, *C. ramiflora* or *M. cylindrocarpa*). All three species fruit during the peak fruiting season, with some low-level fruiting throughout the year. *Aglaia mariannensis* and *M. cylindrocarpa* are fleshy fruited. We chose to centre the sites on three of the most abundant tree species in the forest because we expected these species to have widespread seed rain due to their high abundance, and thus, differences in the seedbank around these species are more likely to reflect differences in dispersal of other species rather than differences in the canopy above each sample.

Plots were fenced during the peak fruiting season between 4 and 6 months before sampling to exclude invasive deer (*Cervus mariannus*) and feral pigs. Deer are thought to be primarily browsers in this system, so are unlikely to affect seed density. Pigs can act as both seed predators and seed dispersers (Sanguinetti and Kitzberger 2010; O'Connor and Kelly 2012), and there is some evidence they may do so in this system (A. Gawel, pers. comm.). Deer and pigs are present on Guam and Rota, but absent or at low densities on Saipan.

We took between 3 and 12 samples from each plot, depending on the heterogeneity of the substrate. Because we expected that the seedbank might vary based on the substrate at a particular microsite, we sampled separately from each of the four primary substrate types: soil, rocky soil, loose karst and solid karst **[see** **Supporting Information****—Text]**. Within each plot, we took three soil samples from each substrate type that comprised at least 20 % of the forest floor. Each sample was separated by at least 1.5 m from a previous sample of the same substrate, but not necessarily a previous sample from a different substrate. If a site contained only 1 substrate, we took up to 3 samples, whereas if it contained all 4 substrates, we took up to 12 samples depending on the availability of each substrate.

We sampled using 0.15 × 0.15 m quadrats because the use of soil cores was not possible on the karst substrate, and soil, when present, rarely exceeded 6 cm in depth. Litter and soil samples were combined for each sample. For bare or loose karst areas, we searched within each quadrat for 5 min, using tweezers to extract seeds where necessary. Where moveable rocks were present on loose karst, we lifted rocks where necessary/possible and searched the area underneath, up to a maximum depth of 6 cm. For rocky soil and soil, we used a trowel to collect all soil and leaf litter up to a maximum depth of 6 cm, or less if bedrock was reached.

In processing samples, all visible seeds were removed from both soil and litter. The remaining soil was sieved to break up any large lumps and searched again. We considered whole, intact seeds as viable, and counted only those seeds. The seeds of herbaceous species and vines were not included, and we focussed only on seeds of tree species. The smallest tree seed within these forests is *Pipturus argenteus* (Urticaceae), measuring 0.64 mm^2^, which is visible by eye. For the genera *Ficus* and *Eugenia*, which both have more than one species present in these forests, seeds were assigned to genus level only. Only one primarily abiotically dispersed tree species was found in our seed samples (*Leucaena leucocephala*); however, the seeds of this species have previously been reported as dispersed by rodents, birds and cattle (Pacific Island Ecosystems at Risk 2012) (Table 1).

**Table 1.** Dispersal syndrome used in analyses for each species of seed recorded in the seedbank on each of the three islands, mean size of seeds and the island on which each species was recorded in the seedbank. The total number of seeds recorded and total number samples taken on each island are given. ^1^A bird dispersal syndrome was assigned based on whether fruits of the species have previously been recorded as eaten by birds or based on the presence or absence of a fruity pulp and the size of a seed: where pulp was present and seeds were small enough to be consumed or carried by the largest vertebrate frugivore that once occurred on the island a species considered to be adapted for vertebrate dispersal (as in Caves *et al*. 2013). ^2^E. Fricke, unpublished data, unless otherwise stated. ^3^Although showing no adaptations for vertebrate seed dispersal, seeds of this species are reported to have been dispersed by rodents, birds and cattle (Pacific Island Ecosystems at Risk 2012). ^4^http://pages.bangor.ac.uk/~afs101/iwpt/web-sp7.htm. ^5^Wiles and Fujita (1992).

Species	Dispersal syndrome^1^	Approx. seed area (mm^2^)^2^	Island		
			Guam	Rota	Saipan
*Aglaia mariannensis*	Bird/bat	182.4	15	2	3
*Aidia cochinchinensis*	Bird	3.3	0	0	1
*Eugenia* spp.	Bird	96.7	27	0	1
*Ficus* spp.	Bird/bat	0.9	0	2	0
*Guamia mariannae*	Bird	89.3	9	0	2
*Leucaena leucocephala*	Gravity/wind^3^	21.0^4^	0	1	194
*Macaranga thompsonii*	Bird	10.6	187	6	0
*Melanolepis multiglandulosa*	Bird	18.3	1	1	20
*Morinda citrifolia*	Bird	35.3	79	0	0
*Ochrosia mariannensis*	Bat^5^	10.1	15	0	2
*Ochrosia oppositifolia*	Bat^5^	354.8	44	0	0
*Carica papaya*	Bird/bat	20.0	0	14	10
*Premna obtusifolia*	Bird/bat	9.2	7	3	67
*Psychotria mariana*	Bird	29.2	0	11	161
	Total number of seeds (total number of samples)		384 (130)	40 (56)	461 (68)

### Distance to conspecific

We tested whether seeds were more likely to have a reproductively mature conspecific (i.e. conspecific adult) nearby in the absence of seed dispersers. We assumed that it would be unlikely for most seeds to arrive via gravity dispersal from a parent tree that is >2 m away, given the low stature of the forest canopy. To identify nearby adult conspecifics likely providing seeds via gravity, we surveyed all adult trees with a canopy overlapping each plot or within a distance of 2 m from the edge of the plot from which the sample was taken. These data were also used to calculate the species richness of the surrounding canopy.

### Analysis

To account for the nestedness of our design, we fitted linear mixed models to our data. We predicted that if frugivorous vertebrates are an important determinant of the distribution and composition of seeds present in forest seedbanks, then we would see differences between Guam and the two islands with dispersers in each of the variables we examined. We, therefore, assessed whether island was an important predictor of variation in each of our response variables, with site included as a random effect in each model. Additional fixed and random effects were assessed or included where relevant, and we detail those for each response variable below. To determine whether the inclusion of seeds from the only potentially wind-dispersed species in the study, *L. leucocephala*, affected each of the qualitative results, we ran analyses both with and without *L. leucocephala*.

We fitted models in R (R Development Core Team 2015) using the package lme4 (Bates and Maechler 2014). We assessed the significance of island as a fixed effect by comparing a model that included it with one that included only an intercept term using likelihood ratio tests. For models fitted using a normal distribution, we assessed the significance of differences between islands using Satterthwaite's approximation for degrees of freedom within the package lmerTest (Kuznetsova *et al*. 2014), with models re-levelled to enable pairwise comparisons between islands. For models fitted using a binomial or Poisson distribution, we assessed these differences using the Wald *Z* test in lme4.

First, we tested the hypothesis that greater seed movement in the presence of frugivores would be associated with a lower proportion of species in the seedbank with a conspecific adult within 2 m. We recorded whether or not (one or zero, respectively) a species recorded in the seedbank at each plot had a conspecific neighbour within 2 m, i.e. we had one value per species per plot. Because multiple species were often recorded within each plot, we included plot as an additional random effect in the model, nesting it within site. By recording the presence of a conspecific neighbour at the level of the species rather than seed, this measure is independent of seed number, which can vary among species. Instead, this measure reflects the proportion of species found in the seedbank that could only have arrived in the seedbank through dispersal. We specified a binomial error distribution.

Second, we tested whether frugivore absence would be associated with a patchy distribution of seeds by examining two response variables. Because we obtained between 3 and 12 samples per plot (depending on substrate), we examined the within-plot variation in the number of seeds per sample. For each plot, we calculated the coefficient of variation (CV) of the total number of seeds per sample. No seeds were recorded in 3 of the 44 plots. We excluded these plots from the analysis as we were specifically interested in testing for within-plot variation in seed density. Data were normally distributed and no transformations were made. In addition, we determined the proportion of samples that contained zero seeds on each island. Here, we specified a binomial error distribution. As multiple samples were taken per plot, we included ‘plot’ as an additional random effect, nesting it within site. We also examined the potential for substrate (which was recorded at the sample level) to influence the proportion of samples that contained seeds by including substrate as a fixed effect in the model and comparing this model with one that did not include substrate as a fixed effect using a likelihood ratio test.

Finally, to examine whether frugivores would increase the small-scale species richness of seeds in the seedbank, we quantified the mean number of species per sample. We examined the potential for the species richness of the surrounding canopy to influence seedbank richness by including the number of adult tree species recorded within 2 m of each plot as a fixed effect in the model, and comparing this model with one that did not include the number of adult tree species as a fixed effect using a likelihood ratio test. Because adult tree species richness was calculated at the plot level, we modelled mean number of species per sample by summing together the number of species recorded in each sample at each plot to give one value per plot (such that if a species was recorded in two samples it would count twice) and offsetting this by the number of samples taken at each plot. We specified a Poisson distribution.

We calculated marginal *R*^2^ values $\left({R_{m}}^{2}\right)$ and conditional *R*^2^ values $\left({R_{c}}^{2}\right)$ for the final model used in each case (Nakagawa and Schielzeth 2013) using the MuMIn R package (Barton 2014). Marginal *R*^2^ values are those due to fixed effects only, while conditional *R*^2^ values are those due to fixed plus random effects.

## Results

Overall, the number of seeds we recorded in samples was low and variable (Table 1). We recorded few seeds of the focal tree species in seedbank samples. As expected in our system, the majority of species we recorded are primarily dispersed by vertebrates, with the exception of one species common on Saipan, *L. leucocephala*.

### Proportion of seeds with a conspecific within 2 m

**Figure 2.**
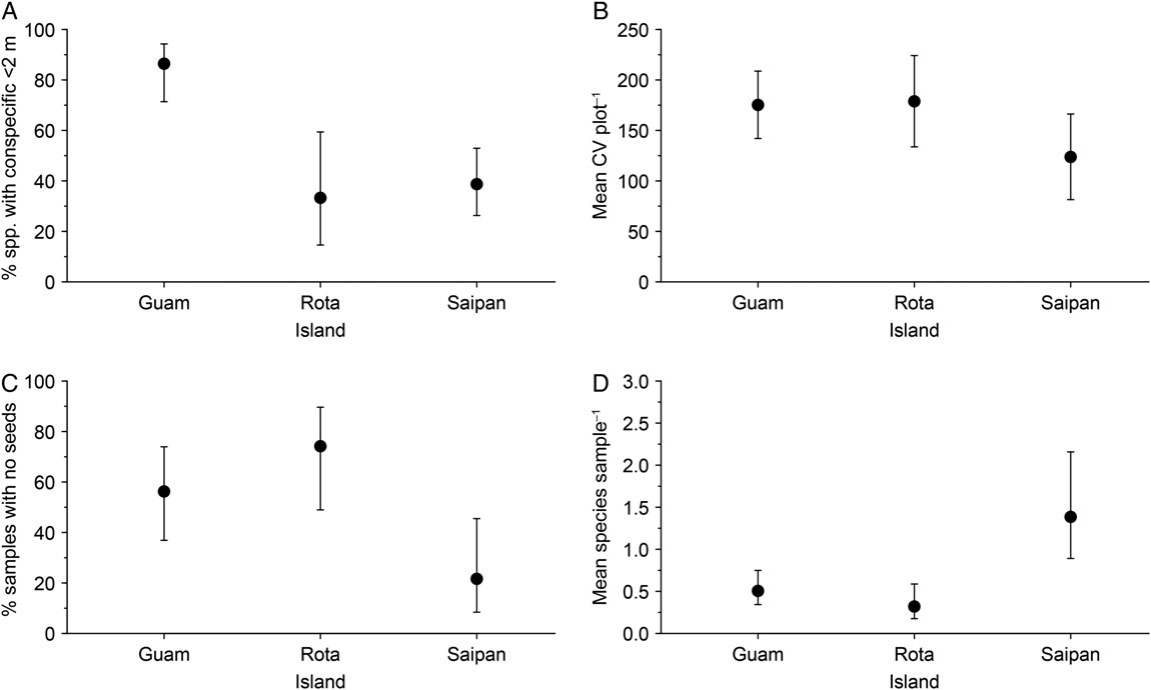
The per cent of species found in the seedbank at each plot that had an adult conspecific within 2 m on Guam where dispersers are functionally absent relative to Rota and Saipan where they are present (A), mean CV in seed density per seedbank sample at each plot (B), the per cent of seedbank samples that lacked any seeds (C) and the mean number of species per seedbank sample (D). Bars represent 95 % confidence intervals.

Island was a significant predictor of the proportion of species in the seedbank with a conspecific adult present within 2 m (*χ*^2^ = 13.86, df = 2, *P* < 0.001, ${R_{m}}^{2}=0.29,$ ${R_{c}}^{2}=0.29$). Species sampled within Guam's seedbank were more likely to have conspecific adults present within 2 m than on both Rota (*z* = −3.50, *P* < 0.001) and Saipan (*z* = −4.11, *P* < 0.001). While 86.5 % of species in the seedbank had a conspecific adult nearby on Guam, this was only true for 33.3 and 38.8 % of species in the seedbank on Rota and Saipan, respectively (Fig. 2A).

### Spatial distribution of seeds within the seedbank

There were no differences between islands in the between-sample variation in seed density (*χ*^2^ = 4.69, df = 2, *P* > 0.05, ${R_{m}}^{2}=0.15,$ ${R_{c}}^{2}=0.20$). Although the CV was lower for samples on Saipan when compared with Guam (Fig. 2B), this was not significant (*t* = −2.19, *P* = 0.05).

Substrate was not an important predictor of the per cent of samples that contained zero seeds (*χ*^2^ = 6.23, df = 3, *P* = > 0.05) and was excluded from the final model. There was a significant influence of island on the per cent of samples that contained zero seeds (*χ*^2^ = 7.36, df = 2, *P* = 0.03, ${R_{m}}^{2}=0.14,$ ${R_{c}}^{2}=0.35$). While more than half of samples on both Guam (56.3 %) and Rota (74.2 %) were devoid of seeds, less than a quarter (21.7 %) of samples on Saipan lacked seeds (Fig. 2C). This difference was significant when comparing Saipan with both Guam (*z* = −2.26, *P* = 0.02) and Rota (*z* = −2.99, *P* = 0.003). However, if we excluded seeds of *L. leucocephala*, the only species we recorded that is primarily wind or gravity dispersed and was found predominantly on Saipan, the difference between Guam and Saipan was no longer significant (*z* = −1.87, *P* = 0.06) **[see** **Supporting Information****—Figure S1]**.

### Species richness of the seedbank

A total of 14 species were recorded in the seedbank across the three islands (Table 1). The species richness of the surrounding canopy was not a significant predictor of the species richness of the seedbank (*χ*^2^ = 0.42, df = 1, *P* > 0.05) and was not included in the final model. The mean number of species per sample was low overall, but again varied between the three islands (*χ*^2^ = 10.55, df = 2, *P* < 0.01, ${R_{m}}^{2}=0.22,$ ${R_{c}}^{2}=0.28$). Saipan had greater species richness than both Guam (*z* = 3.36, *P* < 0.001) and Rota (*z* = 3.85, *P* < 0.001) with 1.39 species per sample, compared with only 0.32 species per sample on Rota and 0.51 species per sample on Guam.

## Discussion

The soil seedbank is an important source of regeneration following disturbance, but our understanding of the processes that determine the distribution and composition of seeds in the seedbank at the community level remains limited. We demonstrate a role of vertebrate frugivores in building and maintaining forest seedbanks. On the island of Guam, where most vertebrate frugivores have been absent for about 30 years, >80 % of seeds in the seedbank were found to have a conspecific adult neighbour. This was in contrast to the seedbank sampled on the nearby islands of Saipan and Rota (which support vertebrate frugivores) where a majority of seeds on the forest floor had no conspecific neighbour and thus were likely dispersed there from >2 m away. In addition, a greater proportion of samples lacked seeds, and the species richness of seedbank samples was lower on Guam relative to Saipan, as predicted if dispersers influence the spatial pattern and diversity of seedbanks. However, we did not find differences in the variability of seed densities or per sample species richness between Guam and Rota, indicating that the presence of dispersers alone is not sufficient to explain these patterns. We hypothesize that high rates of post-dispersal predation may be responsible for the reduced density and richness of seeds in the seedbank on Rota. Since seedbanks strongly reflect the surrounding trees when dispersers are lost from a system, patterns of forest regeneration are unlikely to be maintained by recruitment from either persistent or transient seedbanks.

### Species in the seedbank escape conspecific adults where dispersers are present

Seed dispersal is considered important for moving seeds away from parent trees. Here, we demonstrate the magnitude of that effect for seeds in seedbanks: while 60–70 % of seeds found in the seedbank on islands with native vertebrate frugivores are likely the result of dispersal, in the absence of dispersers, >80 % of seeds are likely from nearby adult trees. This pattern in the seedbank is mirrored by seedling communities in other defaunated sites around the world, where the seedlings closely reflect the identity of the nearby adults (Terborgh *et al*. 2008; Harrison *et al*. 2013). The potential impact of recent declines in some frugivorous bird species on Rota (Camp *et al*. 2014) was not evident in our study, as a similar per cent of seeds in the seedbanks on Rota as on Saipan likely arrived through vertebrate dispersal (i.e. did not have a conspecific adult within 2 m). The failure of seeds present in the seedbanks on Guam to escape their parent plants could have implications for the role of the seedbank in forest regeneration because seeds landing in close proximity to conspecifics are predicted to experience an increase in distance-dependent mortality (Dalling *et al*. 1998; Kotanen 2007).

### Seed dispersal results in a more even distribution of seeds

Seed dispersal is not only important for moving seeds away from parent plants but also for reducing density-dependent mortality by redistributing seeds within the landscape (Comita *et al*. 2014). Without dispersal, seeds should fall in higher densities underneath parent trees and fail to reach sites away from parent trees, leading to greater variation in seed density across the landscape. However, we found no evidence that seed density per sample was more variable on Guam than on other islands, although more samples lacked seeds on Guam than on Saipan.

We have identified four possible reasons for the increase in seedless areas on Guam relative to Saipan. First, we expect that more seeds would experience density- or distance-dependent mortality when not being moved away from conspecifics. High distance-dependent mortality associated with proximity to parent trees may explain why few seeds of the three target tree species were recorded in our sample. An alternative explanation is that seeds from the focal species may not persist in the seedbank, as is common with larger-seeded species (Hopkins and Graham 1983). Second, if seed longevity in the seedbanks is low across all species, which might be expected given the shallow soils and moist conditions of these islands, the seedbank in the absence of dispersal would only contain seeds if it is within close proximity to a tree that has recently reproduced. Third, the pattern could be driven by the presence of a non-native wind-dispersed tree species, *Leucaena*, which is more common on Saipan. However, while the difference between Saipan and Guam is no longer significant when *Leucaena* seeds are omitted, the trend that Saipan has fewer samples without seeds remains. Finally, post-dispersal process such as seed predation may vary across islands, which we discuss in more detail below.

Counter to our predictions, the seedbank on Rota more closely resembled that on Guam in terms of the proportion of samples that lacked seeds, and overall seed abundance (Table 1). Since there appears to be adequate dispersal on Rota, with >65 % of seeds coming from trees >2 m away, we hypothesize that post-dispersal seed predators are responsible. The two most likely post-dispersal predators on Rota are the Malayan black rat (*Rattus rattus diardii* Jentink) and the Cuban slug. Rat densities are similar between Rota and Saipan and higher on both those islands than on Guam (Wiewel *et al*. 2009), indicating that seed predation by rats is unlikely the reason for the varying seed densities between Saipan and Rota. Instead, it is possible that the Cuban slug (*Veronicella cubensis* L. Pfeiffer), a seed predator that is considered a major pest on Rota but is only present in low abundance on Guam and Saipan (Robinson and Hollingsworth 2004; H. S. Rogers, unpubl. data), is responsible for this change. The limited seedbank present in Rota could, therefore, indicate the potential for post-dispersal seed predation to decrease the role of the seedbank in forest regeneration. If so, further work will be needed to tease apart the relative contribution of seed dispersal and seed predation to the spatial distribution of seedbanks.

### Seed dispersal promotes the species richness of the seedbank

Although the movement of seeds away from parent trees and reduction of distance- and density-dependent mortality are generally considered key mechanisms through which the species richness of forests is maintained (Harms *et al*. 2000; Mangan *et al*. 2010), the importance of biotic seed dispersal in structuring forest communities is still often overlooked. One reason for this is that when dispersers are lost from a system, the impacts on adult tree communities may not be seen for several decades (Terborgh 2013) or even centuries (Kelly *et al*. 2004). However, recent evidence demonstrates an impact of disperser loss on the species composition of the seedling community (Terborgh *et al*. 2008; Effiom *et al*. 2013; Harrison *et al*. 2013), a finding that highlights the importance of biotic seed dispersal in structuring forest communities over even short timescales. Our finding that the seedbank has fewer species not present in the surrounding canopy and lower species richness in the absence of vertebrate frugivores suggests that biotic seed dispersal has an important role in determining the species composition of soil seedbanks. Since the early regeneration of forests following disturbance is expected to start with the seedbank (Chandrashekara and Ramakrishnan 1993; Grombone-Guaratini and Rodrigues 2002), we would expect any influence on the species composition of the seedbank to translate to the regenerating seedling community.

## Conclusions

The seedbank is thought to be important for storing seeds until the conditions are right for germination, and for facilitating early regeneration after disturbance (Grime 1989; Grombone-Guaratini and Rodrigues 2002). In that way, seedbanks may have a role in buffering forests against short-term losses in seed input (Thompson 2000). By comparing the seedbanks on islands with and without seed dispersers, we demonstrate a role of seed dispersal in structuring forest seedbanks. We show that seed dispersal is important for moving seeds away from adult conspecifics and maintaining the species richness of the seedbank. These findings not only highlight the importance of dispersers for building and maintaining forest seedbanks, but suggest another mechanism by which current global declines in vertebrate assemblages could have important implications for forest persistence.

## Sources of Funding

Funding was provided by a United States National Science Foundation award DEB-1258148 to H.S.R., A.E.D., R.H.M. and H.S.R. was also supported by a Huxley Fellowship from the Department of BioSciences at Rice University.

## Contributions by the Authors

E.M.W., H.S.R., A.E.D. and R.H.M. conceived the idea. E.M.W. and H.S.R. collected the data and carried out the analyses. E.M.W. led the writing with assistance from H.S.R., A.E.D. and R.H.M.

## Conflict of Interest Statement

None declared.

## Supporting Information

The following additional information is available in the online version of this article –

**Table S1.** Lists bird species present or previously present on Guam, Rota and Saipan.

**Text.** Description of the four substrate types present in the forests of each island.

**Figure S1.** Results of each analysis when *Leucaena leucocephala*, the only species predominantly dispersed by wind or gravity, is excluded.

Additional Information
