# Supplementary material for: Vertebrate seed dispersers maintain the composition of tropical forest seedbanks
Source: AoB Plants. 2015 Nov 17;7:plv130. doi: 10.1093/aobpla/plv130 (PMC4689121; doi:10.1093/aobpla/plv130)
Supplement: Additional Information [file supp_7_plv130_index.html]

Vertebrate seed dispersers maintain the composition of tropical forest seedbanks — Additional Information 

# Vertebrate seed dispersers maintain the composition of tropical forest seedbanks

## Additional Information

Additional Information

- Additional Information - Docx file
