# Supplementary material for: Vertebrate seed dispersers maintain the composition of tropical forest seedbanks
Source: AoB Plants. 2015 Nov 17;7:plv130. doi: 10.1093/aobpla/plv130 (PMC4689121; doi:10.1093/aobpla/plv130)

**FILE 1.**

TABLE. Frugivorous bird species present, or previously present on the three islands studied: Guam, Rota and Saipan. P (present) indicates that a species is currently present on that island, E (extinct) indicates that the species was previously present but is now extinct, A (absent) indicates that the species has never been present on that island.

**Species Island**

**Guam Rota Saipan**

Micronesian Starling (*Aplonis opaca*) P P P

Mariana Fruit Dove (*Ptilinopus roseicapilla)* E P P

White-throated Ground Dove *(Gallicolumba xanthonura)* E P P

Bridled White-eye (*Zosterops conspicillatus*) E P P

Rota Bridled White-eye (*Zosterops rotensis*) A P A

Mariana Crow (*Corvus kubaryi*) E P A

**FILE 2.** Description of the four main substrate types identified.

**Soil**: Fine-grained material with few/no obvious karst chunks present.

**Rocky soil:** Smaller (< ~ 5 cm) pieces of karst are discernible within a matrix of soil. Few to no large chunks of karst are present.

**Loose karst:** large karst chunks (>5 cm) dominate but the substrate is not solid rock.

**Solid karst:** a solid piece of karst, such as a large boulder or a karst pinnacle.

**FILE 3.**

FIGURE. Values for each response variable examined when seeds of *Leucaena leucocephala*, the only species dispersed primarily by wind or gravity, are excluded: the percent of species found in the seedbank at each plot that had an adult conspecific within 2 m on Guam where dispersers are functionally absent relative to Rota and Saipan where they are present (A), mean CV in seed density per seedbank sample at each plot (B), the percent of seedbank samples that lacked any seeds, which is no longer significantly different between Saipan and Guam (*P* > 0.05, C) and the mean number of species per seedbank sample (D). Bars represent 95% confidence intervals.


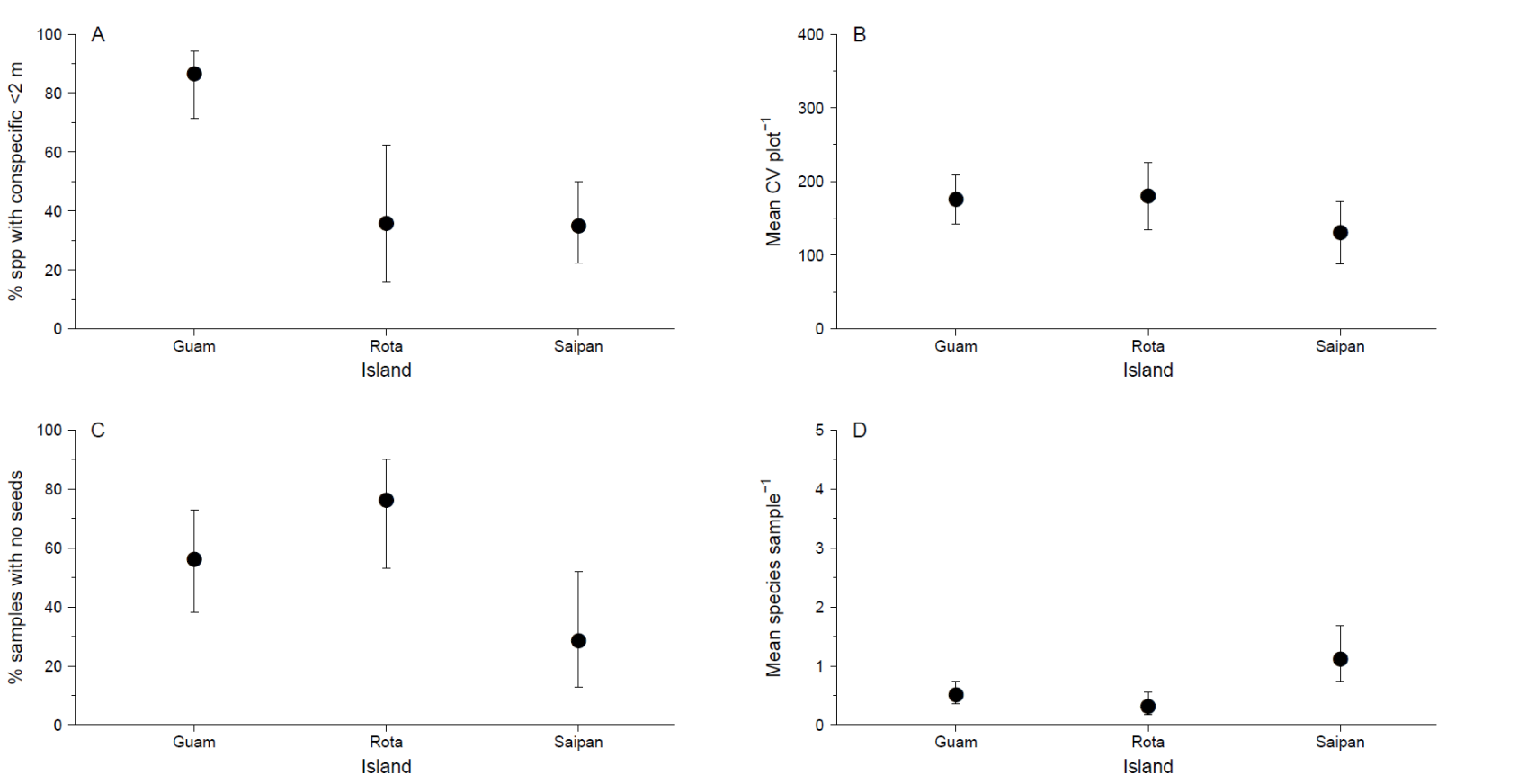

Supplement: Additional Information [file supp_plv130_plv130supp.docx]
